# Fractal-Based Radiomic Approach to Tailor the Chemotherapy Treatment in Rectal Cancer: A Generating Hypothesis Study

**DOI:** 10.3389/fonc.2021.774413

**Published:** 2021-12-09

**Authors:** Carmela Di Dio, Giuditta Chiloiro, Davide Cusumano, Francesco Catucci, Luca Boldrini, Angela Romano, Elisa Meldolesi, Fabio Marazzi, Barbara Corvari, Brunella Barbaro, Riccardo Manfredi, Vincenzo Valentini, Maria Antonietta Gambacorta

**Affiliations:** ^1^ UOC Radioterapia Oncologica, Mater Olbia Hospital, Olbia, Italy; ^2^ Dipartimento Diagnostica per Immagini, Radioterapia Oncologica ed Ematologia, Fondazione Policlinico Universitario Agostino Gemelli IRCCS, Rome, Italy

**Keywords:** radiomics, MRI, oxaliplatin, rectal cancer, predictive modeling

## Abstract

**Introduction:**

The aim of this study was to create a radiomic model able to calculate the probability of 5-year disease-free survival (5yDFS) when oxaliplatin (OXA) is or not administered in patients with locally advanced rectal cancer (LARC) and treated with neoadjuvant chemoradiotherapy (nCRT), allowing physicians to choose the best chemotherapy (CT) regimen.

**Methods:**

LARC patients with cT3–4 cN0 or cT1–4 cN1–2 were treated according to an nCRT protocol that included concomitant CT schedules with or without OXA and radiotherapy dose of 55 Gy in 25 fractions. Radiomic analysis was performed on the T2-weighted (T2-w) MR images acquired during the initial tumor staging. Statistical analysis was performed separately for the cohort of patients treated with and without OXA. The ability of every single radiomic feature in predicting 5yDFS as a univariate analysis was assessed using the Wilcoxon–Mann–Whitney (WMW) test or t-test. Two logistic models (one for each cohort) were calculated, and their performance was assessed using the area under the receiver operating characteristic (ROC) curve (AUC).

**Results:**

A total of 176 image features belonging to four families (morphological, statistical, textural, and fractal) were calculated for each patient. At the univariate analysis, the only feature showing significance in predicting 5yDFS was the maximum fractal dimension of the subpopulation identified considering 30% and 50% as threshold levels (maxFD_30–50_). Once the models were developed using this feature, an AUC of 0.67 (0.57–0.77) and 0.75 (0.56–0.95) was obtained for patients treated with and without OXA, respectively. A maxFD_30–50_ >1.6 was correlated to a higher 5yDFS probability in patients treated with OXA.

**Conclusion:**

This study suggests that radiomic analysis of MR T2-w images can be used to define the optimal concomitant CT regimen for stage III LARC cancer patients. In particular, by providing an indication of the gross tumor volume (GTV) spatial heterogeneity at initial staging, maxFD_30–50_ seems to be able to predict the probability of 5yDFS. New studies including a larger cohort of patients and external validation sets are recommended to verify the results of this hypothesis-generating study.

## Introduction

Colorectal cancer is one of the most widespread cancer diseases in the world, causing the death of thousands of people each year, as recently estimated in the latest epidemiological studies (1, 2).

The standard treatment for locally advanced rectal cancer (LARC) consists of neoadjuvant chemoradiotherapy (nCRT) followed by surgery (1, 3).

Recent experiences demonstrated that local control (LC) can be improved by the combination of nCRT and surgery, but no significant benefit has been observed in terms of disease-free (DFS) and overall survival (OS) (4). Furthermore, it has been observed that the 25% of patients develop metastases within 5 years after the end of the surgery, mainly located in the liver (5).

In this context, the intensification of nCRT could be a valid strategy. In recent years, several studies have been conducted to evaluate the efficacy of adding oxaliplatin to nCRT; however, the benefit of oxaliplatin-based nCRT in stage II or III rectal cancer remains unclear. Several randomized trials investigated the effect of oxaliplatin-based nCRT: efficacy data are controversial, and the addition of oxaliplatin often resulted in increased acute toxicity (6–11). For this reason, the role of oxaliplatin is still a matter of debate, especially in patients with stage III rectal cancer.

Radiomics is playing a primary role in proposing new image-based markers that can predict surrogate endpoints of survival outcomes such as pathological complete response (pCR) or DFS in order to personalize neoadjuvant treatment (12–14). These predictors are generally based on MR image analysis, as it is the gold standard for diagnosis and staging of rectal cancer (12, 15, 16).

First experiences proposing radiomic models able to predict different outcomes, such as OS or metastasis-free survival, have recently been reported in the literature (17, 18).

Although the potential of radiomics in extracting prognostic factors from image analysis is now widely accepted by the scientific community, to the best of our knowledge, there are no predictive models in the literature that support the oncologist in deciding which drug to prescribe for neoadjuvant treatment.

In this experience, we want to explore the potential of radiomics in drug personalization, proposing an MRI-based indicator able to predict the DFS probability at 5 years after the end of treatment (5yDFS) with a high level of accuracy in two cohorts of patients (one in which oxaliplatin was administered and one in which it was not administered).

In particular, the final goal of this hypothesis-generating study is to obtain a model that can calculate the probability of 5yDFS for both oxaliplatin administration and non-administration, allowing clinicians to choose the best chemotherapy (CT) regimen based on the highest probability of 5yDFS.

## Materials and Methods

### Patients’ Selection Criteria and Treatment Workflow

Patients enrolled in this retrospective study were affected by locally advanced rectal adenocarcinoma, with cT3–4 cN0 or cT1–4 cN1–2 or with mesorectal fascia involvement (MRF+), according to the American Joint Committee on Cancer (AJCC) TNM (19).

Treatments were delivered from May 2008 to June 2015 at Fondazione Policlinico Universitario Agostino Gemelli IRCCS in Rome. Ethics committee approval was obtained for this study, and all patients gave signed informed consent to be enrolled. At the time of diagnosis, patients had to be older than 18 years with pathologically confirmed rectal adenocarcinoma; cases with mucinous variants were excluded from the study.

All selected LARC patients received nCRT followed by surgery. Patients with missing treatment information, with metastatic disease at diagnosis, and alive patients without evidence of local or distant recurrence with a follow-up time less than 5 years were excluded.

MRI safety screening forms were administered to all patients: those who showed clinical contraindications to MRI or denied specific consent were considered not eligible for the study. Clinical and radiological follow-up was performed for all the patients for a period of at least 5 years after surgery.

For tumor staging, MRI acquisition was performed using a 1.5-T scanner (Signa Excite, GE Medical Systems, Milwaukee, WI, USA) in the supine position. The MRI protocol consisted of four T2-weighted fast spin-echo (FSE) MR sequences (axial, coronal, sagittal, and volumetric) and one diffusion-weighted imaging (DWI) acquisition obtained using b values of 0 and 1,000 s/mm^2^. No intravenous contrast agents were administered.

Radiotherapy (RT) treatment was delivered in 25 fractions, following a simultaneous integrated boost (SIB) technique with 55 Gy in fractions of 2.2 Gy/die to gross tumor volume (GTV) and corresponding mesorectum and 45 Gy in fractions of 1.8 Gy/die to selected lymph nodes according to the clinical disease (20). Neoadjuvant CT was administered according to two regimens based on initial clinical stage and patient compliance:

- *OXA-based regimen:* CapOx (60 mg/m^2^ of i.v. oxaliplatin at the first day plus 1,300 mg/die/mq of oral chronomodulated capecitabine 1,650 mg/mq/die, during RT q7) or Xelox (oxaliplatin 130 mg/mq q 1, 19, and 38 plus oral chronomodulated capecitabine 1,300 mg/mq/die during RT).
- *No-OXA-based regimen:* 5-fluorouracil in continuous infusion (225 mg/mq/die during RT) or oral chronomodulated capecitabine (1,650 mg/mq/die during RT).

At 6 to 10 weeks after the end of nCRT, patients underwent clinical restaging, consisting of a restaging MRI and digital rectal examination. Surgery was performed 8 to 12 weeks after the end of nCRT and consisted of abdominal-perineal resection, anterior resection, or transanal endoscopic microsurgery, depending on the residual disease and surgical evaluation.

The postoperative histopathological specimen was classified following the tumor regression grading (TRG) according to the Mandard classification (21).

Adjuvant CT was administered for selected patients in relation to clinical and pathological stages and high-risk factors such as tumor lymph vascular invasion and TRG4.

### Radiomic Analysis

Radiomic analysis was performed on the T2-weighted FSE MR images acquired in a transverse plane orthogonal to the tumor longitudinal axis during initial disease staging (22). The MR images subject to radiomic analysis were those acquired during the initial tumor staging.

Such images had a spatial resolution of 0.8 × 0.8 mm^2^ and a slice thickness of 3 mm, with no intersection gap between the slices. Repetition time ranged from 2,500 to 5,000 ms, inversion time from 100 to 110 ms, and echo train length from 16 to 24.

The Dicom files containing the MR images were imported into an RT delineation console (Eclipse, Varian Medical System™, Palo Alto, CA, USA) where a radiologist in cooperation with a radiation oncologist experienced in rectal cancer diagnosis and treatment delineated the GTV, following the ICRU n.83 guidelines (23).

Delineations were blinded between the two experts, and a final consensus was discussed and obtained with a shared delineation in case of disagreement. Dicom files containing MR images and contours were exported and processed using Moddicom, an R package (R Core Team, Vienna, Austria) designed to perform radiomic analysis of biomedical images (24, 25).

Two image filters were applied to the original MR images before extracting radiomic features: the Laplacian of Gaussian (LoG) filter with a kernel size dimension of 0.4 mm and the intensity-based (IB) image filter, with a step of 20%: additional information about the use of these filters can be found in (26, 27).

**Figure 1:**
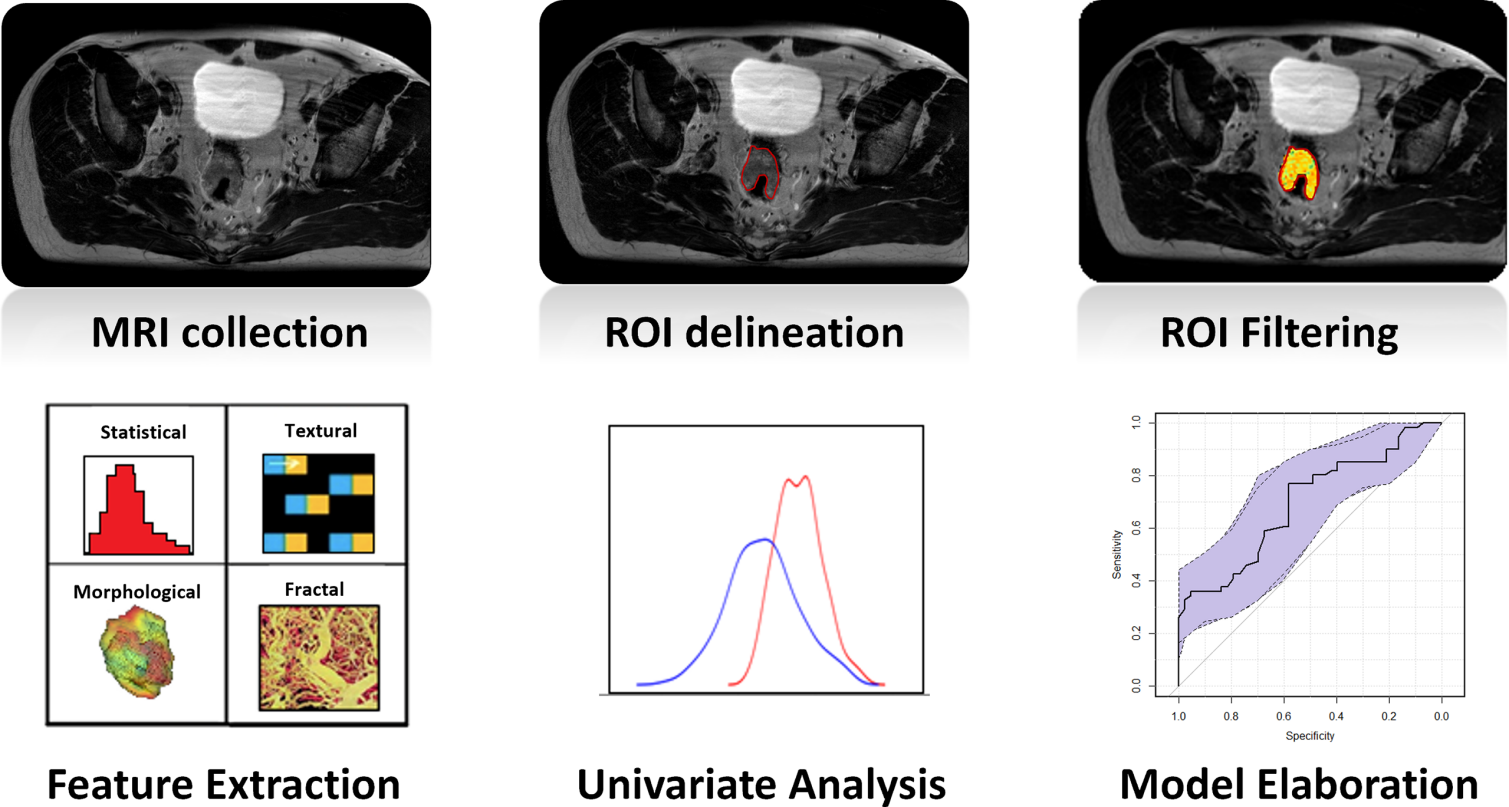
Diagrammatic representation of the whole radiomic process: once MR images were collected, GTV was delineated on each MRI. Images were filtered, and four types of radiomic features (statistical, textural, morphological, and fractal) were extracted. The ability of every single feature in predicting the outcome was evaluated in terms of the Wilcoxon–Mann–Whitney test, and a logistic regression was calculated considering the most significant feature. The last two steps were repeated separately for the two cohorts of patients of the study (OXA and no-OXA). GTV, gross tumor volume; OXA, oxaliplatin.

Radiomic analysis led to the extraction of four feature families: morphological features were extracted from the raw images, statistical and textural features were extracted from the MR images preprocessed using the LOG filter, fractal features were extracted from the images processed with the IB filter (27, 28). A diagrammatic representation of the whole radiomic process is reported in **Figure 1**.

As regards the textural features, three gray-level matrices were considered: run length (rlm), co-occurrence (cm), and size zone (szm) matrices. The complete list of the radiomic features extracted is reported in the Supplementary Materials, with similar experiences dealing with this topic (15, 29).

Fractal features were instead calculated on the images preprocessed using the IB filter, which consists in normalizing the pixel values within GTV using the first and 99th percentiles of the gray-level GTV histogram as extremes and then identifying pixel clusters based on different threshold levels defined as the percentage of the maximum intensity (27, 30).

Once the images were processed, fractal dimension (FD) was calculated slice by slice using the Box counting algorithm, and minimum, maximum, median, and mean values were calculated and considered as fractal features (27).

### Statistical Analysis

Statistical analysis was performed separately for the cohort of patients treated with (OXA-based) and without oxaliplatin (no-OXA-based), considering that the DFS reached 5 years from the date of surgery (5yDFS) as a dichotomic outcome.

**Figure 2:**
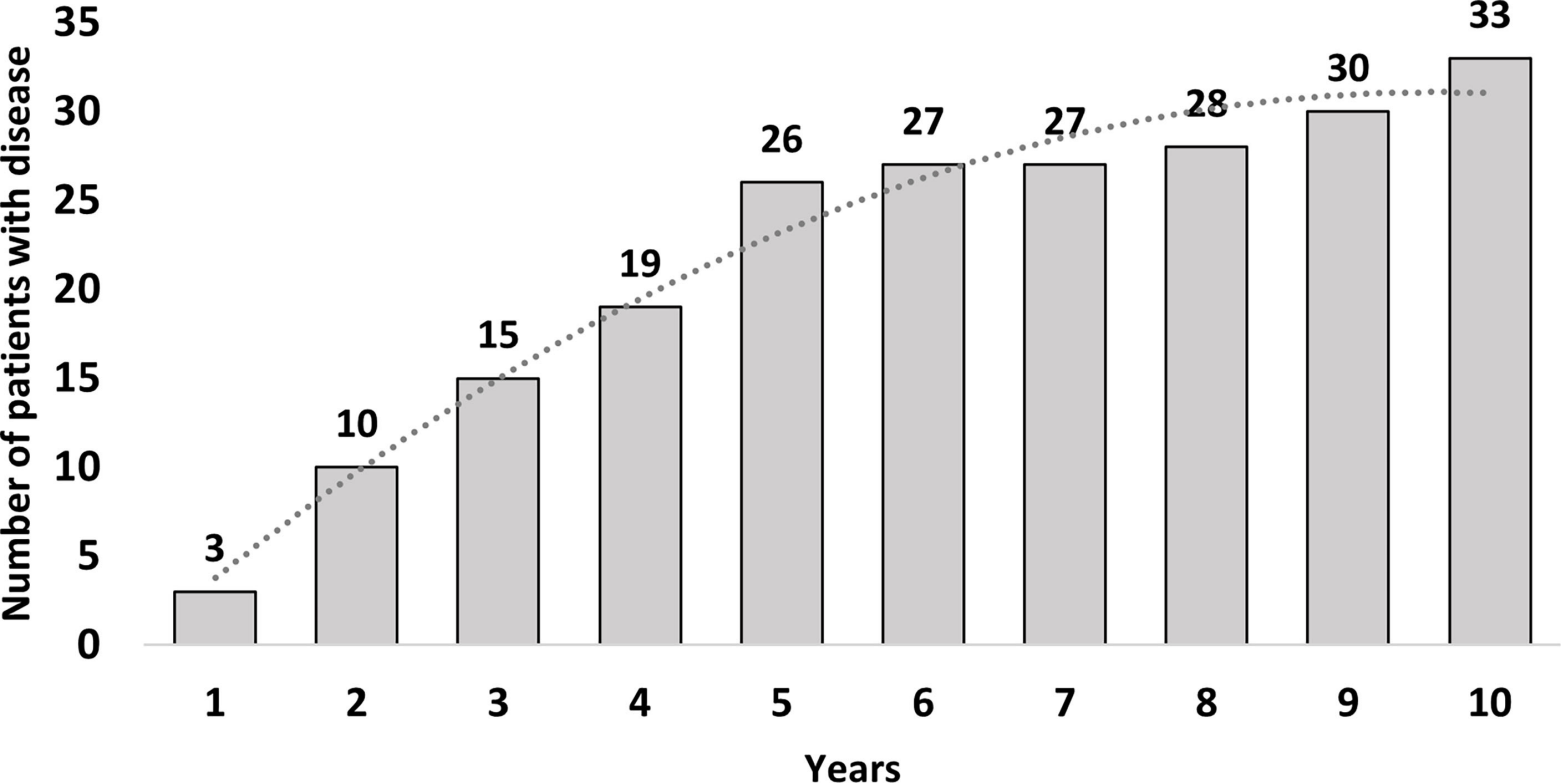
Number of patients showing metastases in relation to the years after the end of surgery.

**Figure 2** reports the number of patients showing metastases or tumor recurrence as a function of the years from the date of surgery: the time of 5 years was chosen as the optimal cutoff between the number of events that occurred and the number of patients with valid follow-up at that time.

The ability of every single radiomic feature in predicting 5yDFS at the univariate analysis was assessed by considering the Wilcoxon–Mann–Whitney (WMW) test or the t-test, depending on the normality of data distribution, which was previously evaluated using the Shapiro–Wilk test (31).

Clinical information such as initial tumor staging, GTV volume, sex, and age at diagnosis was considered as an additional variable. The radiomic feature showing the highest level of significance in both the patient cohorts was considered as the best predictor, and two logistic regression models were developed, one for each cohort.

The predictive performance of the two predictive models was evaluated using the area under the receiver operating characteristic (ROC) curve (AUC), with the 95% CIs calculated using the bootstrap method with 2,000 iterations (32).

The best cutoff threshold was identified maximizing Youden’s index (J), and the sensitivity and specificity values at the best threshold were calculated for each model (33).

With the use of the elaborated models, the probability of 5yDFS at different values of the radiomic parameter was calculated to identify different ranges in which a CT regimen can be considered of higher value, ensuring a higher probability of 5yDFS.

The robustness of the two developed models was evaluated by means of a threefold cross-validation analysis with five iterations, as an independent external validation dataset was not available (30).

The whole statistical analysis was performed using R software (version 3.6.1, Wien Austria) and dedicated packages (34).

## Results

A total of 240 patients were considered for this study, but only 188 cases met the inclusion criteria: 125 (66%) were treated with oxaliplatin CT and 63 (34%) without oxaliplatin CT. The clinical characteristics of the patients included in the study are summarized in **Table 1**.

**Table 1:** Clinical characteristics of patients enrolled and treated with and without oxaliplatin.

Clinical characteristics	OXA-based regimen n = 125	No-OXA-based regimen n = 63
**Age (range)**	61 (26–81)	64 (32–83)
**Sex**		
Male	83 (66%)	38 (58%)
Female	42 (34%)	25 (42%)
**Clinical tumoral stage**		
cT2	3 (2%)	9 (14%)
cT3	72 (58%)	45 (72%)
cT4	49 (40%)	9 (14%)
**Clinical nodal stage**		
cN0	3 (2%)	10 (16%)
cN1–2	122 (88%)	53 (84%)
**MRF involvement**		
Positive	47 (38%)	50 (79%)
Negative	78 (62%)	13 (21%)
**GTV (cm^3^) (range)**		
Pre	47.92 (30.21–210.53)	25.28 (24.02–76.22)
Post	18.16 (7.47–120.49)	8.74 (4.47–64.40)

Categorical variables are reported with the percentage of evidence, continuous variable with mean values, and corresponding range.

OXA, oxaliplatin; GTV, gross tumor volume; MRF+, mesorectal fascia involvement.

The median follow-up time was 96 months, with a range of 9–156 months, and with a 5yDFS of 84.8% and 87.3% for OXA- and no-OXA-based CT, respectively.

A total of 176 image features (92 radiomic and 84 fractal features) were extracted for each patient: among the radiomic features, 14 were based on morphology, 22 on gray-level histogram analysis (1st-order features), and 46 on the textural analysis (16 szm, 18 rlm, and 22 cm).

At the univariate analysis, the only feature that showed statistically significant ability in predicting 5yDFS in both patient cohorts was the maximum FD of the subpopulation identified considering 30% and 50% as threshold levels (maxFD_30–50_), with a p-value of 0.018 in the cohort treated with oxaliplatin and 0.019 in the one treated without oxaliplatin. The values of the parameters and coefficients characterizing the two models developed are reported in **Table 2**.

**Table 2:** Covariates and coefficients of the linear logistic regression models elaborated to predict 5yDFS from the analysis of T2-w MR staging images.

Regimen	Covariate	Coefficient	Sigma coefficient	p-Value
OXA-based	Intercept	19.43	7.57	0.01
	maxFD_30–50_	−10.91	4.62	0.02
No-OXA-based	Intercept	22.15	10.15	0.03
	maxFD_30–50_	−12.67	6.25	0.04

5yDFS, 5-year disease-free survival; T2-w, T2-weighted; OXA, oxaliplatin.

**Figure 3:**
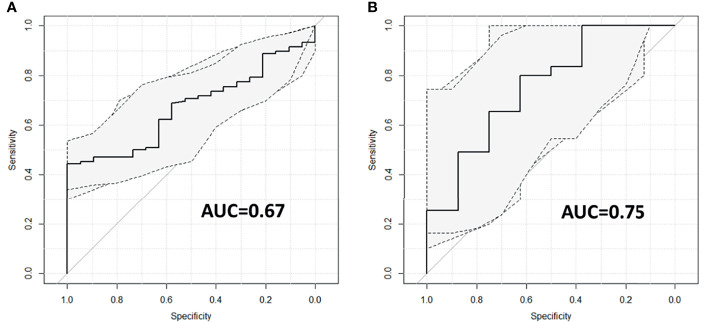
ROC curves with corresponding 95% CIs for patients treated with **(A)** and without **(B)** oxaliplatin. ROC, receiver operating characteristic.

**Figure 3** reports the ROC curves with the corresponding 95% CIs obtained for the two logistic regression models created using maxFD_30–50_ as variable and 5yDFS as the outcome.

For the OXA-based cohort, the predictive model reports an AUC of 0.67 (95% CI ranging from 0.57 to 0.77); for the no-OXA-based cohort, the model shows an AUC of 0.75 (95% CI ranging from 0.56 to 0.95). The best cutoff threshold was 0.88 (corresponding to a J index of 0.44) for patients on OXA-based regimen, 0.80 (J = 0.42) for patients in no-OXA-based regimen. At the best threshold value, the sensitivity was 44.3% for patients treated with an OXA-based regimen and 80% for patients treated with no-OXA regimen, while the specificity was 100% and 62.5%, respectively. The robustness analysis performed using the threefold cross-validation reported an AUC of 0.67 with an SD of 0.06 for the oxaliplatin cohort and an AUC of 0.75 with SD equal to 0.15 for the no-oxaliplatin cohort.

**Figure 4:**
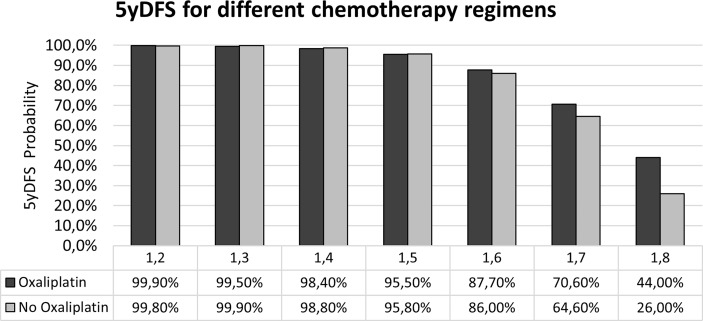
Probability of 5yDFS to varying of the maxFD_30–50_ extracted from the T2-w staging MR images in the case of the two treatment regimens. 5yDFS, 5-year disease-free survival; T2-w, T2-weighted.

Applying the two models developed, the probability of achieving 5yDFS when oxaliplatin is or is not administered can be calculated. **Figure 4** summarizes the values obtained in terms of 5yDFS probability to varying maxFD_30–50_ values.

**Figure 5:**
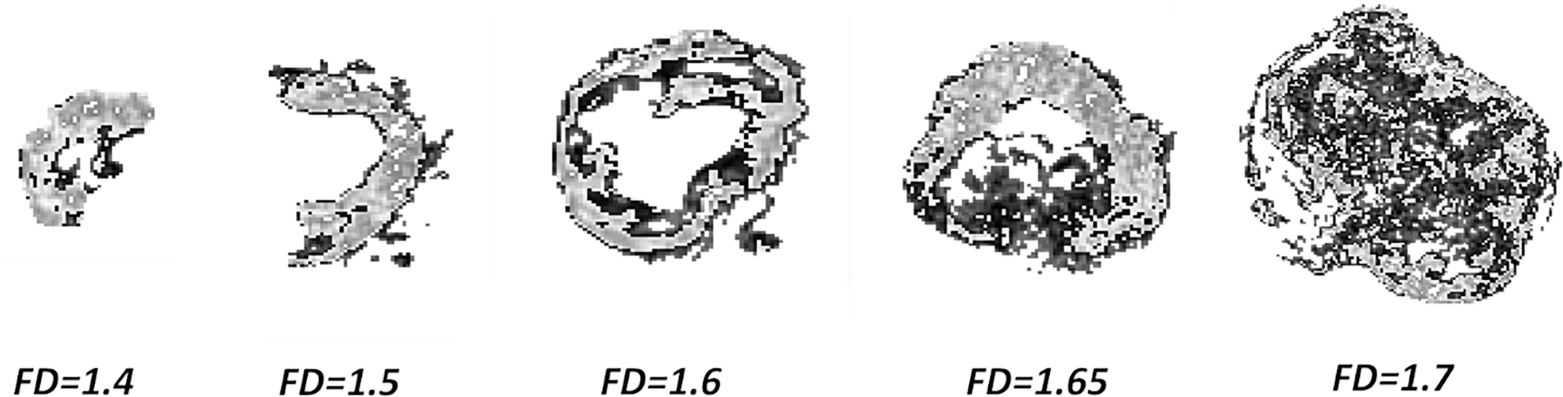
Complexity of GTV to increasing maxFD_30–50_ values: pixels in light gray indicating the subpopulation between 30% and 50% of the maximum intensity. GTV, gross tumor volume.

**Figure 5** reports a visual representation of the meaning of FD: such parameter can be considered as a metric indicator of the tumor aggressiveness, as higher FD values describe tumor structures characterized by a more complex spatial arrangement. In particular, the higher value of maxFD_30–50_ is correlated with a lower probability of 5yDFS.

## Discussion

During recent years, the number of radiomic applications has exponentially grown, demonstrating that the image-based biomarkers can play a significant role in the context of the omics-based predictive models, at the same level as more advanced disciplines such as genomics, proteomics, and transcriptomics (35–37).

This work represents one of the first experiences that aim to identify the role of radiomics in the definition of a more intensive schedule of concomitant CT at the time of diagnosis, in order to reduce the rate of local and distant metastases at 5 years.

For rectal cancer, indeed, the majority of the experiences are focused on predicting early surrogate endpoints, such as the pCR, because these data are more quickly available in a clinical setting and allow a faster analysis of the radiomic potentialities (30, 38, 39).

Developing models that can predict long-term outcomes is much more challenging, as it requires a very precise and time-consuming follow-up analysis: one of the strengths of this experience is that it is based on the analysis of patients with a very long follow-up (median of 96 months).

Interestingly, an innovative methodology was proposed in this experience, with the aim of identifying the optimal CT regimen for every single patient, based on initial staging MRI analysis.

Patients were divided into two cohorts, and the features able to predict 5yDFS with statistical significance in both the cohorts were analyzed: the only one showing predictive ability in both the cohorts was the FD, which is an interesting point, as previous experiences in rectal cancer had demonstrated the fractal potentialities in predicting pCR from staging MRI analysis (27, 30).

In the idea of applying the proposed model in clinical reality, the therapeutic choice would be based on the maxFD_30–50_ value: an oxaliplatin-based regimen should be preferred in cases of maxFD_30–50_ >1.6, as it ensures a higher 5yDFS probability (70.6% vs. 64.6% in case of FD = 1.7; 44% vs. 26% in cases of FD = 1.8), while the two CT regimens can be considered equivalent in terms of 5yDFS for maxFD_30–50_ ≤1.6.

Obviously, the findings of this study are still not mature enough to be considered ready for clinical applications, as they are not supported by an external validation: to partially compensate for such absence, internal cross-validation was performed, which had confirmed the results observed in the training set.

Furthermore, the 95% CI values of the ROC curves elaborated are still quite large, mainly due to the small number of events analyzed: a more comprehensive study including larger patient cohorts is recommended to verify the potential of FD as an image-based biomarker in rectal cancer.

In conclusion, this study proved the feasibility of establishing the optimal regimen of CT combined with nCRT for stage III LARC cancer patients based on information extracted from the analysis of T2-w MR images. In particular, by providing an indication of the spatial heterogeneity of GTV at staging, maxFD_30–50_ is able to predict with statistical significance the probability of 5yDFS. New studies including a larger cohort of patients and external validation sets are recommended to verify the results of this hypothesis-generating study.

## Data Availability Statement

The raw data supporting the conclusions of this article will be made available by the authors, without undue reservation.

## Ethics Statement

The studies involving human participants were reviewed and approved by Gemelli. The patients/participants provided their written informed consent to participate in this study. Written informed consent was obtained from the individual(s) for the publication of any potentially identifiable images or data included in this article.

## Author Contributions

All authors contributed to the article and approved the submitted version.

## Conflict of Interest

The authors declare that the research was conducted in the absence of any commercial or financial relationships that could be construed as a potential conflict of interest.

## Publisher’s Note

All claims expressed in this article are solely those of the authors and do not necessarily represent those of their affiliated organizations, or those of the publisher, the editors and the reviewers. Any product that may be evaluated in this article, or claim that may be made by its manufacturer, is not guaranteed or endorsed by the publisher.
